# QUALITATIVE FINDINGS AND THEMES IN REIMAGINING LTC: RESULTS ROUND 1 AND 2 OF THE NATIONAL DELPHI STUDY

**DOI:** 10.1093/geroni/igz038.2336

**Published:** 2019-11-08

**Authors:** Rosalie A Kane, Audrey Workman

**Affiliations:** 1 DIvision Health Policy & Management, School of Public Health. University of Minnesota, Minneapolis, Minnesota, United States; 2 U of MN School of Public Health, Minneapolis, Minnesota, United States

## Abstract

From Round 1 we developed programmatic building blocks, which we classified as: housing suggestions; services suggestions; housing and/or technology heavy suggestions; policy or regulation suggestions;new philosophical approaches; and long-range social engineering. Besides the quantitatively ratings of the importance of each building block, respondents explained what they liked and disliked about each.. They frequently commented that environments rich in design features, amenities and activities would not be practical for low-income people. Respondents felt that some ideas would not be suitable for people with dementia because they would be insufficiently protected. Principles that seemed to be incompatible could be highly endorses; e.g., the principle that we prioritize people staying in their own homes and a principle that frail elderly persons living along should relocate to group residential settings to avoid social isolation. This paper concludes with a list of areas for further discussion by work groups.

